# Prediction of pathogenicity genes involved in adaptation to a lupin host in the fungal pathogens *Botrytis cinerea* and *Sclerotinia sclerotiorum* via comparative genomics

**DOI:** 10.1186/s12864-019-5774-2

**Published:** 2019-05-17

**Authors:** Mahsa Mousavi-Derazmahalleh, Steven Chang, Geoff Thomas, Mark Derbyshire, Phillip E. Bayer, David Edwards, Matthew N. Nelson, William Erskine, Francisco J. Lopez-Ruiz, Jon Clements, James K. Hane

**Affiliations:** 1 UWA School of Agriculture and Environment, The University of Western Australia, 35 Stirling Highway, Crawley, WA 6009 Australia; 2 Centre for Crop and Disease Management, School of Molecular and Life Sciences, Curtin University, Bentley, WA 6102 Australia; 3 Department of Primary Industries and Regional Development, 3 Baron-Hay Court, South Perth, WA 6151 Australia; 4 School of Biological Sciences, The University of Western Australia, 35 Stirling Highway, Crawley, WA 6009 Australia; 5 UWA Institute of Agriculture, The University of Western Australia, 35 Stirling Highway, Crawley, WA 6009 Australia; 6 Natural Capital and Plant Health, Royal Botanic Gardens Kew, Wakehurst Place, Ardingly, West Sussex, RH17 6TN UK; 7 Curtin Institute for Computation, Curtin University, Bentley, WA 6102 Australia; 8 Current address: Agriculture and Food, Commonwealth Scientific and Industrial Research Organisation, Wembley, WA 6913 Australia

**Keywords:** *Sclerotinia sclerotiorum*, *Botrytis cinerea*, Lupin, Comparative genomics

## Abstract

**Background:**

Narrow-leafed lupin is an emerging crop of significance in agriculture, livestock feed and human health food. However, its susceptibility to various diseases is a major obstacle towards increased adoption. *Sclerotinia sclerotiorum* and *Botrytis cinerea* – both necrotrophs with broad host-ranges - are reported among the top 10 lupin pathogens. Whole-genome sequencing and comparative genomics are useful tools to discover genes responsible for interactions between pathogens and their hosts.

**Results:**

Genomes were assembled for one isolate of *B. cinerea* and two isolates of *S. sclerotiorum*, which were isolated from either narrow-leafed or pearl lupin species. Comparative genomics analysis between lupin-derived isolates and others isolated from alternate hosts was used to predict between 94 to 98 effector gene candidates from among their respective non-conserved gene contents.

**Conclusions:**

Detection of minor differences between relatively recently-diverged isolates, originating from distinct regions and with hosts, may highlight novel or recent gene mutations and losses resulting from host adaptation in broad host-range fungal pathogens.

**Electronic supplementary material:**

The online version of this article (10.1186/s12864-019-5774-2) contains supplementary material, which is available to authorized users.

## Background

Narrow-leafed lupin (*Lupinus angustifolius* L.) is an important grain legume crop of south and west Australia and Northern Europe which significantly contributes to animal feed, aquaculture and sustainable farming because of its nitrogen fixation and use in rotation systems [1]. In recent years lupin has been also promoted as a human health food, being gluten-free, high in protein and dietary fibre, and low in starch and fat [2]. Pearl lupin (*L. mutabilis* Sweet., Andean Lupin, tarwi) is a minor indigenous crop in South America for which limited breeding work has been carried out [3]. Although the lupin industry has been successful in Western Australia and some European countries, such as Poland, Russia and Germany, susceptibility to various diseases prevents it reaching maximum potential [4]. Two diseases known to impact lupin health are Sclerotinia stem rot and grey mould, caused by the fungi *Sclerotinia sclerotiorum* and *Botrytis cinerea*, respectively [5].

Both *S. sclerotiorum* and *B. cinerea* are necrotrophic Ascomycetes with broad host-ranges [6]. *S. sclerotiorum* infects more than 400 hosts, including several lupin species [7]. *S. sclerotiorum* used to be an intermittent disease of lupin, however increased occurrence on canola (*Brassica napus*) - with which it is often rotated - may have led a recent corresponding rise in lupin [8]. Trials have indicated that fungicide application on lupins infected by *S. sclerotiorum* only marginally reduces disease [8]. Similarly, *B. cinerea* has been ranked second in a recently compiled list of the most important fungal plant pathogens [9], infecting more than 200 plant species and causing severe pre- and post-harvest damage worldwide.

Identifying fungal gene products that promote host-infection is important for improving disease management. These include small-secreted protein (SSP) effectors and secondary metabolites (SM) [10, 11]. Increased availability of whole-genome sequence resources of plant pathogenic fungi have allowed bioinformatic prediction of effector-like proteins [12] which can then be targeted in the development of durable disease resistance strategies [13]. The genomes of *S. sclerotiorum* and *B. cinerea* are of high quality representations of whole chromosomes, supported by long-read sequencing and optical or genetic maps [14, 15]. These genomes share significant sequence similarity and conserved synteny, but differ significantly in their repetitive DNA content and repertoire of SSPs and SM synthesis genes [6]. Genome-based studies in *Sclerotinia* and *Botrytis* have been instrumental in the identification of several putative effectors that may be associated with virulence [14, 16, 17]*.* Comparative genomics of other broad host-range species, such as the *Colletotrichum* spp. (*C. sublineola* and *C. graminicola*) have also predicted several non-conserved effector-like SSPs and SM proteins with potential roles in virulence [18], despite overall high levels of genome sequence conservation. Collectively, these studies have utilized genomic variations between different species to highlight genes that may be relevant to host-pathogen interactions; however it appears that useful variations can also be detected across isolates of a single species. In the broad host-range pathogen *Coleosporium ipomoea,* it has been postulated that isolates may narrow in host range towards host-specificity as they co-evolve with their respective hosts [19].

In this study, we outline and compare genome sequences for two isolates of *S. sclerotiorum* isolated from *L. angustifolius* and *L. mutabilis*, and one isolate of *B. cinerea* from *L. angustifolius*. The development of genomic resources specific to fungal pathogens of lupin will lay the foundations for further improvements on genome-driven integrated disease management of this crop. Furthermore, identification of variable genes between very recently diverged isolates of the same species, may provide insight into recent adaptations that are a result of challenge from defences of differing hosts, region-specific environmental conditions, farming practices or disease controls.

## Results

### Genome features

Genome assembly of paired-end Illumina reads - with raw coverage of approximately 81x in *S. sclerotiorum* isolated from *L. angustifolius* (subsequently referred to as Sscl-Lang), 164x in *S. sclerotiorum* isolated from *L. mutabilis* (Sscl-Lmut) and 90x in *B. cinerea* isolated from *L. angustifolius* (Bcin-Lang) - resulted in 796 sequences with a total length of ~ 38.40 Mb for Sscl-Lang and 1091 contigs with a total length of length of ~ 38.44 Mb for Sscl-Lmut. These genomes are predicted to encode 12,196 proteins in Sscl-Lang and 12,146 proteins in Sscl-Lmut (Table 1). The Bcin-Lang assembly produced a total length of ~ 41.97 Mb, present in 216 sequences and encoding 13,353 proteins (Table 1). CEGMA [20] analysis showed a high percentage of highly conserved core eukaryotic genes were present in all three draft assemblies with 95.97% in Sscl-Lang, 97.18% in Sscl-Lmut and 96.37% in Bcin-Lang. Proteins from these genomes were functionally annotated with gene ontology (GO) terms assigned to 4925 (40.38%) and 4922 (40.46%) of predicted proteins of Sscl-Lang and Sscl-Lmut respectively and Pfam domains assigned to 7025 (57.60%) and 6995 (57.59%) genes in Sscl-Lang and Sscl-Lmut respectively. In Bcin-Lang, 5422 (40.60%) and 7772 (58.20%) of genes were assigned GO terms and Pfam domains respectively. The count of genes with assigned Pfam domains was compared between isolates/species using Fisher’s exact test (*P* ≤ 0.05) (Additional file 1). This analysis showed that there was variation in gene content at the functional level between isolates collected from different hosts. Gene-based information for all the isolates are provided in Additional file 2. Repeat content of these genomes were highly similar (Additional file 3). De novo prediction of repeat sequences predicted 6.32, 6.46, and 2.53% of the Sscl-Lang, Sscl-Lmut and Bcin-Lang assemblies as repetitive, while prediction based on comparison to the known fungal repeats in Repbase predicted 2.32, 2.38, and 1.7% (Table 1).

**Table 1:** Isolate information (A), genome (B) and predicted gene set statistics for lupin-infecting isolates of *Sclerotinia sclerotiorum* and *Botrytis cinerea* compared to reference isolates

	*Sclerotinia sclerotiorum*	*Sclerotinia sclerotiorum*	*Sclerotinia sclerotiorum*	*Botrytis cinerea*	*Botrytis cinerea*
A) Isolate information					
Isolate abbreviation	Sscl-Lang	Sscl-Lmut	Sscl-Ref	Bcin-Lang	Bcin-Ref
Isolate ID	–	–	1980 UF-70	WAC9891	B05.10
Isolated from (host species)	*Lupinus angustifolius*	*Lupinus mutabilis*	*Phaseolus vulgaris*	*Lupinus angustifolius*	Unknown
Isolated from (region)	Western Australia	Western Australia	Nebraska, USA	Western Australia	Germany
B) Genome					
Total assembly length (Mbp)	38.40	38.44	38.81	41.98	42.90
Maximum length (Mbp)	1.1	0.7	3.9	1.7	4.1
N50 length (Mbp)	0.2	0.2	2.4	0.6	2.4
Total sequences	796	1091	16	216	18
GC%	41.5	41.5	41.5	42.0	42.0
%Repetitive (de novo)	6.3	6.5	9.5	2.5	4.4
%Repetitive (Repbase:“Fungi”)	2.3	2.4	4.2	1.7	3.1
C) Predicted gene set					
CEGMA % (complete | partial)	95.97 | 98.39	97.18 | 99.19	–	96.37 | 98.79	96 | 98.8
Total number of predicted proteins	12,196	12,146	11,368	13,353	11,701
Exons / Gene	3	3	–	2.9	–
Introns / Gene	2	2	–	1.9	–
Proteins with GO annotation	4925 (40.4%)	4922 (40.5%)	5745 (50.5%)	5422 (40.6%)	–
Proteins with Pfam annotation	7025 (57.6%)	6995 (57.6%)	7298 (64.2%)	7772 (58.2%)	–

A survey of AT-rich regions in these genomes, which is a common signature of repeat-induced point mutations (RIP) [21], revealed little evidence of RIP in Sscl-Lmut and only one gene in Sscl-Lang that corresponded to an AT-rich region. In Bcin-Lang, 22 genes were associated with AT-rich regions (Additional file 4). However, none of the above genes were predicted to be effector candidates (see below) and only four out of 22 had Pfam domain associated with them. The lengths of these genes ranged from 154 to 19,641 bp (Additional file 4). Summary results of sub-cellular localization of proteins are presented in Additional file 2. Carbohydrate active enzyme (CAzyme) complements of Bcin-Lang, Sscl-Lang and Sscl-Lmut were investigated and the most abundant CAzymes in all three pathogens were Glycoside Hydrolases (GHs) classes.

### Prediction of effector genes in Sscl-Lang, Sscl-Lmut and Bcin-Lang

Putative effector genes were predicted using the intersect of EffectorP and SignalP predictions, and were then compared with databases of known pathogenicity factors DFVF [22] and PHI-base [23] (Additional files 2 and 5). This resulted in identification of 98 candidate effector proteins in Bcin-Lang, 94 in Sscl-Lang and 96 in Sscl-Lmut (Additional file 5). Pfam domain assignments were not common among these candidates, however the most commonly assigned was “fungal hydrophobin” (PF06766). Using the same approach, 80 and 74 candidate effector genes were also predicted in reference isolates of *B. cinerea* B05.10 and *S. sclerotiorum* 1980-UF, respectively, which were used in subsequent presence-absence variation (PAV) analysis (Additonal file 4, see below).

### Presence/absence variation

**Fig. 1:**
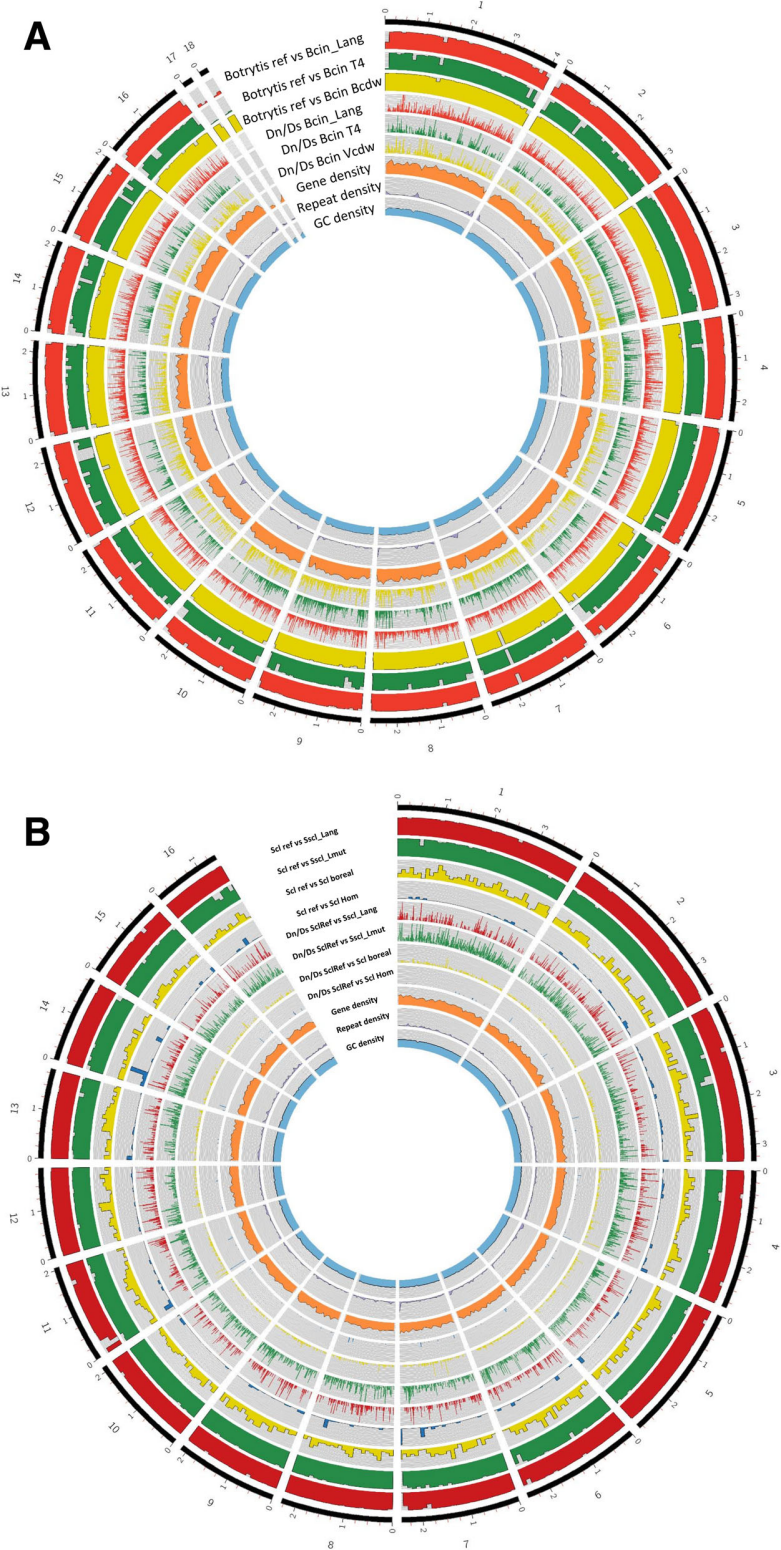
Genome features, mutation profiles and presence-absence variations across isolates of *Botrytis cinerea* and *Sclerotinia sclerotiorum*. Chromosomes of reference isolates *Botrytis cinerea* B05.10 (**a**) and *Sclerotinia sclerotiorum* 1980-UF (**b**) are visualised alongside (from the innermost ring outwards): genome features including G:C content, gene and repeat densities; ratios of non-synonymous to synonymous mutations (Dn/Ds) relative to alignments of lupin-infecting isolates over 100 kb intervals, and the percent of 100 kb regions that aligned to lupin-infecting isolate assemblies. Yellow boxes indicate large regions of absence in the reference isolate relative to lupin-infecting isolates (Additional file 2)

Sequence conservation analysis showed distinct regions of PAV across Sscl-Lang, Sscl-Lmut and *Sclerotinia* spp. Similar patterns were also identified between Bcin-Lang and *Botrytis* spp. (Fig. 1). Isolate-specific genes were identified by reference alignment (Additional file 2, Additional file 6) and by orthology (Additional file 4, Table 2). We found one gene (*bcinT_12260*) in Bcin-Lang that was missing from reference isolate which was associated with the DnaJ domain (PF00226).

**Table 2:** Count of variable genes belonging to predicted functional categories across lupin-infecting isolates. Genes unique to lupin-infecting isolates were grouped into functional categories predicted by SignalP, EffectorP or Pfam (Additional file 2). Isolate-specificity was determined within sequences with predicted presence-absence variation (PAV) relative to their respective reference isolate, or by non-orthology (N-O) between the gene sets of isolates of the same species (Additional file 4)

Species	*Botrytis cinerea*		*Sclerotinia sclerotiorium*			
Isolate abbreviation	Bcin-Lang		Sscl-Lang		Sscl-Lmut	
Method	PAV	N-O	PAV	N-O	PAV	N-O
Count of Signal P (secreted)	17	98	0	10	0	18
Count of signal p with Effector P (score > 0.8)	2	22	0	5	0	3
Protein repeat motifs (PF00023, PF13606, PF12796, PF00132, PF13374, PF13424, PF00400, PF07676, PF13637)	1	18	0	9	0	11
Membrane transport (PF00664, PF00005, PF13520, PF01544, PF07690, PF01566, PF00083, PF05653, PF01061, PF01554, PF13246)	3	16	0	2	0	2
Domains of unknown function (PF11807, PF11905, PF05670, PF10067, PF07350, PF11913, PF12013, PF01424, PF02656, PF11917, PF12520, PF16297, PF17107, PF06726, PF14087, PF13054, PF04577, PF11327, PF12051, PF12311)	4	9	0	5	0	6
Transposon-related (PF00075, PF03732, PF07727, PF05699, PF00078, PF03221, PF03184, PF13358)	0	6	1	7	1	0
Bacteriophage-related (PF05840, PF02925, PF02305, PF02306, PF04687, PF04726)	6	6	5	0	5	0
Transcription factors (PF00172, PF04082, PF11951, PF00319, PF00170)	4	12	0	0	0	1
Cytochrome P450 (PF00067)	3	14	0	1	0	2
Methyltransferase (PF01035, PF13489, PF13649, PF00891, PF12847, PF08242, PF08241)	0	9	0	0	0	3
Heterokaryon incompatibility (HET) (PF06985, PF14479)	0	10	0	4	0	1
Secondary metabolite synthesis (PF00550, PF03936, PF11991, PF04820, PF08659, PF14765)	0	7	0	0	0	3
Transcriptional regulation (PF14420, PF05712, PF05368, PF03226)	4	7	0	0	0	1
Ubiquitinilation (PF12861, PF14570, PF13639, PF00240, PF00179, PF01485, PF01088, PF09792)	1	7	0	0	0	1
Zinc-binding dehydrogenase (PF00107, PF13602)	1	5	0	0	0	4
Protein kinase domain (PF00069)	0	1	0	1	0	2
NB-ARC domain (PF00931)	0	1	0	9	0	0
AAA family (PF13086, PF13087, PF00004, PF13191)	1	7	0	1	0	1
Glycosyl hydrolase (PF16499, PF00933, PF03659, PF04616, PF01532, PF00295, PF00840, PF00150, PF00332)	2	4	0	0	0	2
FAD binding/metabolism (PF01494, PF01266, PF08022, PF01565, PF00970)	1	5	0	0	0	1
Acetyltransferase (PF00698, PF07247, PF00583, PF13508)	1	4	0	1	0	2
Alpha/beta hydrolase (PF12697, PF07859, PF00561)	2	4	0	0	0	0
Intronic endonuclease (PF13391, PF07453, PF07460, PF14529)	1	2	0	3	0	0
NAD binding/metabolism (PF13450, PF13460, PF00146, PF01370, PF00724)	3	3	0	0	0	0
Beta-ketoacyl synthase (PF00109,PF02801)	0	2	0	0	0	1
Alcohol dehydrogenase GroES-like domain (PF08240)	0	4	0	0	0	2
Dynamin family (PF00350)	1	5	0	0	0	0
NACHT domain (PF05729)	0	0	0	4	0	2
GMC oxidoreductase (PF00732, PF05199)	0	4	0	1	0	0
Carboxyleterase (PF00135)	1	4	0	0	0	0
Coenzyme A metabolism (PF00441, PF07993, PF02771, PF02515)	0	4	0	0	0	0
Enoyl-(Acyl carrier protein) reductase (PF13561)	1	3	0	0	0	0
Tannase and feruloyl esterase (PF07519)	0	4	0	0	0	0
AMP-binding enzyme (PF00501)	0	3	0	0	0	0
DnaJ domain (PF00226)	1	2	0	0	0	0

## Discussion

We report the genome assemblies of two isolates of *S. sclerotiorum* (Sscl-Lang and Sscl-Lmut) and one isolate of *B. cinerea* (Bcin-Lang), isolated from Western Australian lupin hosts. In comparison of these regionally-diverged isolates, that may have also potentially undergone some level of host-specific adaptation, several genome features are expected to vary. First among these we expect background variations in repetitive DNA contents due to their separate evolutionary histories. If host-specific adaptation has also occurred, then we also expect variation in metabolic enzymes involved in degradation of host tissues and in gene content with roles in pathogenicity. In testing for variation in gene content we have employed three methods in parallel: 1) enrichment analysis for functional annotations; 2) PAV analysis of gene orthologs; and 3) PAV of large regions of DNA.

### Comparison of general genomic features

The new genome assemblies of the lupin-infecting isolates appear to contain similar gene contents but differ in repetitive content compared to their respective reference isolates. The genome length of Bcin-Lang was 41.9 Mb, nearly 1 MB smaller than the gapless genome of the *B. cinerea* B05.10 reference isolate [15]. Both assemblies contained similar levels of highly-conserved eukaryotic genes (98.80% vs 98.79% in Bcin-Lang) but differ in repeat content (4.40% vs 2.53%in Bcin-Lang). The Sscl-Lang and Sscl-Lmut genomes were similar in length (38.404 Mb and 38.444 Mb, respectively) and shorter than the *S. sclerotiorum* 1980 reference isolate (38.806 Mb), with fewer repetitive regions predicted (6.32 and 6.46% respectively, vs 9.5% in the 1980 reference). The number of predicted genes in Bcin-Lang was 13,353, which is higher than in B05.10 (11,701) [15]. Sscl-Lang and Sscl-Lmut were also predicted to have more genes (12,196 and 12,146, respectively) compared to the 1980 reference isolate (11,130 [14]). The observed variation in the genome size, repeat content, and number of predicted genes is most likely due to differences between a near-complete reference and highly-fragmented short-read assemblies. The lack of isolate-specific RNA sequencing data to guide gene prediction in this comparative study may have also been a factor, however this was offset by the use of reference isolate RNA-seq and gene annotations to assist gene prediction in these novel isolates.

Regions of sectional gene absence in reference genomes (see methods), yielded a handful of effector candidate genes, with one specific to the *B. cinerea* reference (*Bcin01p03900*), and six (*Bcin12p06110, Bcin12p06760, Bcin13p00040, Bcin04p06980, Bcin08p00020, Bcin09p07100*) conserved across all isolates (including Bcin-Lang) except T4 (Additional file 2, Additional file 6). No effector candidates were found in regions of sectional gene absence for comparisons of *S. sclerotiorum* however a putatively-secreted cerato-platanin gene (*sscle_11g081570*) was identified as conserved in both the *S. sclerotiorum* reference and Sscl-Lmut, but absent from Sscl-Lang. This family of proteins have been recently demonstrated to be important for the virulence of *Sclerotinia sclerotiorum* on *Arabidopsis* and *Nicotina benthamiana* [24, 25]. Within sectional PAVs, other functional annotations were sparse and those identifiable were most commonly cytochrome P450s, CAZymes, transcription factors or protein binding functions (Additional file 2, Additional file 6), with no clear indication of their potential roles in host-range adaptation. We also observed all genes of accessory chromosomes 17 and 18 of the *B. cinerea* B05.10 reference isolate [15] were absent in both the Bcin-Lang and T4 isolates, although their functional roles and consequences of their loss have yet to be determined [26]. For gene orthologs present/absent in lupin isolates we found a gene in Bcin-Lang (*bcinT_12260*) that was missing from the *Botrytis* reference, which was associated with the chaperone DnaJ/Hsp40 family of proteins (Table 1). Studies on *Ustilago maydis* (maize pathogen) and *Fusarium oxysporum* (tomato pathogen) suggest that some members of this family may have roles in virulence [27]. Further investigation is needed to identify the possible role of this chaperone protein in virulence of Bcin_Lang.

The repeat contents of regions of the *S. sclerotiorum* and *B. cinerea* reference isolates exhibiting PAV (by MUMmer) relative to the lupin-infecting isolates were analysed. We observed *S. sclerotiorum* to have repetitive sequences in ~ 12.5–14.1% of the reference genome exhibiting PAV with lupin-infecting isolates, compared to ~ 4% in its conserved regions (Additional file 7). Conversely, we observed *B. cinerea* to have similar proportions of repetitive sequences in both PAV and conserved regions at ~ 4%. This may potentially indicate a relationship between repetitive DNA and variable genome regions which are related to host-adaptation in *S. sclerotiorum* but not *B. cinerea*.

### Genome features involved in plant pathogenicity

Carbohydrate-degrading enzymes are involved in the metabolic breakdown of host cell components during infection [28]. CAZyme profiles were highly similar between isolates versus the references (Additional file 8) [29]. However one gene (*bcinT_03819*) was present only in Bcin-Lang and predicted to have GH43 (glycosyl hydrolase), CBM1 and CBM6 (carbohydrate-binding module) activities, of which CBM6 was not predicted for any of the reference isolate proteins. CBM1 and CBM6 proteins are usually observed to have cellulose−/xylan-binding activities, with the former almost never found in non-Fungi.

RIP is a mutagenesis process specific to some fungi, which in some cases may play an important role in the evolution of pathogenicity-related genes or genome regions [30, 31]. Regions rich in AT bases are typically signatures of RIP, which can be identified within fungal genomes along with genes that are associated with them [21]. Previously the *S. sclerotiorum* 1980 reference isolate (isolated from *Phaseolus vulgaris*) was observed to contain negligible AT-rich content [14, 21]; however, we observed Sscl-Lang (isolated from *L. angustifolius*) had 0.88% AT-rich content, which although very low was predicted as distinct from the rest of the genome by OcculterCut. This analysis identified a single AT-associated gene *SLangusT_02752* that was also unique to that isolate but of unknown function and was not in our candidate effector list. The AT-rich proportion of Bcin-Lang was higher at 4.78% proportion than in genomes of alternate isolates B05.10 (0.932%), T4 (3.6%) and BcDW1 (4.62%) [21]. Bcin-Lang AT-rich regions were associated with 22 predicted genes (Additional file 4), of which five genes (*bcinT_12252*, *bcinT_12257*, *bcinT_13074*, *bcinT_13259*, *bcinT_13332*) were isolate-specific (identified as PAV) with no orthologs in the reference genome. None of these genes were predicted to encode candidate effector proteins. However, one gene (*bcinT_13074*) had a blast match to DFVF (e-value = 5.00E-04) to a putative pathogenicity-related ABC transporter protein of *Magnaporthe grisea* (Q3Y5V5_MAGGR). Out of these 22 genes, four (*bcinT_05697*, *bcinT_05698*, *bcinT_11190*, *bcinT_12252*) were assigned Pfam domains of unknown function, reverse transcriptase, and tannase/feruloyl esterase activity (PF07727, PF07519 and PF12013).

The various isolates of *Botrytis* and *Sclerotinia* appear to have small differences in pathogenicity-related gene content. It may be possible that differences in the relative number of genes grouped by functional annotation may reveal adaptations specific to each isolate that may relate to pathogenicity, environment or response to disease controls. Comparison of functions showed several were over-represented in Bcin-Lang, Sscl-Lang and Sscl-Lmut compared to other isolates, and also to relevant groups of pathogens including legume- and dicot-infecting (Additional file 1). Heterokaryon incompatibility gene (HET), involved in determining the compatability of anastomosis and genetic transfers between cells [32], was more abundant in Bcin-Lang compared to *B. cinerea* B05.10 isolate [6] (*P* = 0.031). A recent study in genetic diversity of 13 different isolates of *B. cinerea* suggested that regions of increased genetic diversity were associated with HET loci [33]. Bcin-Lang and Sscl-Lang genomes also encoded a large number of cytochrome P450 proteins [34] and Bcin-Lang, Sscl-Lang and Sscl-Lmut were enriched in fungal specific transcription factor domain (PF04082) versus their respective reference isolates. NB-ARC domain proteins, which are usually associated with non-self recognition [35], were enriched in Sscl-Lang. However the biological roles of the above functional domains are broad and obscure [33, 34, 36] and the selection pressures they are geared towards would be experimentally challenging to characterise, but we assume that they are likely to have non-pathogenic roles.

Fungal effector proteins are employed by some plant pathogens to promote host colonisation, prevention of host defence responses or otherwise altering host physiology, and also influence pathogen lifestyle and host specificity [37]. Hence, identifying effector repertoires within pathogen genomes and determining their functions is an important step towards developing durable resistance in plants [38]. Several studies have previously computationally predicted effector proteins in *Botrytis* and *Sclerotinia* [6, 14, 16]. We predict 98 effector proteins in Bcin-Lang, out of which 22 were unique (orthology-based) to that isolate. For Sscl-Lang and Sscl-Lmut, 5 and 3 isolate-specific effector candidates were predicted from 94 and 96 predicted effector candidates respectively. None of the isolate-specific effector candidates were assigned functional annotations or matched known pathogenicity factors, with the exception of *SLmutabT_10471* which matched a putative pathogenicity gene from *Blumeria graminis* [DFVF: Q00639_BLUGR] (Additional file 5). Overall, these subtle differences may indicate small variations in gene content between the isolates, which might be relevant to the process of adaptation of broad host range pathogens to a specific host over time.

## Conclusion

In this study, isolates of the broad host-range pathogen species *Sclerotinia sclerotiorium* and *Botrytis cinerea* that were isolated from *Lupinus* spp. were compared to isolates from alternate hosts. With the novel isolates originating in Western Australia from a lupin host, the *B. cinerea* B05.10 reference isolate from an unknown host in Germany, and the *S. sclerotinia* 1980 UF-70 isolate from a *Phaseolus* host in Nebraska, USA [6], these isolates diverged sufficiently to enable bioinformatic detection of numerous sequence variations. Comparisons of isolate genomes revealed minor differences in gene content and/or sequence, some of which may be related to pathogenesis. Overall we observed high levels of similarity, with minor variations such as in repetitive DNA or AT-rich region content, and gene functions unrelated to pathogenicity. Some of these observations are confounded by variable qualities and completeness of genome assemblies, particularly in repeat-rich regions, and lack of supporting data from RNAseq or additional isolates. However among the pool of variable genes that we observed, a small pool of effector-like candidates were predicted, which present interesting opportunities for future analyses. We conclude that comparative genomics can be usefully applied to the predictive analysis of host-specific pathogenicity mechanisms at an intra-species or inter-isolate level in broad host-range fungal pathogens.

## Methods

### Isolate sampling and genomic DNA extraction

Genomic DNA of two isolates of *S. sclerotiorum* were obtained from *L. angustifolius* (Western Australia, Department of Primary Industries and Regional Development (DPIRD)) and *L. mutabilis* (Mt. Barker, 2007, DPIRD), and one isolate of *B. cinerea* was isolated from *L. angustifolius* (South Perth, 1994, DPIRD, WAC9891). Samples were collected by and used in this study with the permission of DPIRD. Seven day-old fungal mycelium was inoculated into 100 mL of half strength potato dextrose broth (PDB), which was incubated at 20 °C in the dark and agitated at 100 rpm for 4 days. Fungal cultures were centrifuged at 10,000 x g for 20 mins and pellets were washed with sterile-distilled water and freeze-dried overnight. Fungal genomic DNA was extracted by the CTAB method [39]. DNA concentrations were quantitated using a Qubit 2.0 fluorometer (Invitrogen, Waltham, MA). For simplicity, we call these isolates Sscl-Lang, Sscl-Lmut and Bcin-Lang, respectively.

### Genome assembly

The whole-genomes of each isolate were sequenced via the Illumina MiSeq platform (Illumina, San Diego, CA), which generated 250 bp paired-end reads from fragments with an average size of 450 bp. Read quality was assessed with FastQC [40], and low quality and adapter sequences were trimmed via Cutadapt v1.9.1 [41] (−-quality-cutoff 30, −-quality-base 33, −-overlap 10, −-times 3 and --minimum-length 25). Overlapping trimmed read pairs were merged into long single-reads via FLASH v1.2.11 [42] (−r 250 -f 400 -s 150). Reads were assembled into contigs via SPAdes version 3.6.1 [43] (default parameters, −careful) and contigs shorter than 200 bp were removed. Contigs were screened for contamination by BLASTN comparison of the NCBI nucleotide database (e-value 1e-4), lowest common ancestor taxa were predicted using MEGAN v5.11.3 [44], and contaminants were discarded as per Additional file 9. Representation of core eukaryotic genes within genome assemblies was estimated using CEGMA [20], as a measure of assembly quality and completeness.

### Gene and repeat feature annotation

Interspersed repetitive DNA sequences were predicted using RepeatMasker [45] to search against both 1) de novo repeats (RepeatModeller: default settings, Repeatmasker:.-lcambig -nolow) and 2) the fungal repeats within the Repbase database (Repbase v, Repeatmasker: -lcambig -nolow –species fungi). Protein and transcript sequences from alternate isolates and closely related fungal species (Additional file 10) were aligned to genome assemblies with the Analysis and Annotation Tool (AAT) (−-dps ‘-f 100 -i 30 -a 200’ --filter ‘-c 10’ --nap ‘-× 10’) [46]. Ab-initio gene predictions were obtained using GeneMark-ES v4 (fungal mode, self-trained) [47] and CodingQuarry [48] (using genes predicted by GeneMark-ES, both normal and pathogen modes). These various evidence types were combined and relatively ranked (GeneMark ab initio predictions = 3; CodingQuarry ab initio predictions = 4; protein alignments = 5; transcript alignments = 7) to produce a reference gene set using EvidenceModeler [49].

### Functional annotation

Functional domains were predicted for gene annotations via Interproscan V5.23–62.0 (Interpro, Pfam, TIGRFAM, TMHMM, SignalP, Phobius, MobiDBLite, Superfamily) [50]. Carbohydrate-active enzyme (CAZyme) annotations were predicted using dbCAN [51] with HMMER v3.0 [52] (default settings). Genes were compared via BLASTP (e-value ≤1e-3) to Swiss-Prot [53] and to databases of known pathogenicity-related proteins: DFVF [22] and PHI-base [23]. Sub-cellular localizations of proteins were predicted using SignalP [54], WoLF PSORT [55] and LOCALIZER [56]. Putative effector-like proteins were predicted via EffectorP [57]. Effector candidates were defined as SSPs – predicted to have a secretion signal peptide by SignalP - with an EffectorP score ≥ 0.8. Genes associated with AT-rich regions were predicted via OcculterCut [21].

### Comparative genomics

Different types of genomic comparisons were made with a view to better understand pathogenic differences between isolates from different hosts. We performed statistical assessment of protein functional attributes, as well as orthology-based functional comparison between lupin infecting and other host-infecting pathogens. The number of genes possessing certain Pfam domains was collected from Integrated Microbial Genomes (IMG/MER) for all published fungal pathogens (as of June 2017), and compared to the functional annotations for the newly sequenced lupin-infecting isolates. Fisher’s exact tests were used to assess statistical significance for over- or -under-representation of functional annotations between Sscl-Lang, Sscl-Lmut, Bcin-Lang, *B. cinerea* B05.10, *S. sclerotiorum* 1980 UF-70 [6] and *S. homoeocarpa* 04–21 [58]. Lupin-infecting isolates were also compared to average counts of relevant groupings of multiple isolates/species, including: lupin-infecting, legume-infecting and dicot-infecting (Additional file 1). We used Proteinortho v5.16 to identify orthologs of Sscl-Lang and Sscl-Lmut compared to predicted proteomes of the *S. sclerotiorum* isolate 1980, [14], then identified non-orthologous genes that were specific to a single isolate. Similarly we compared Bcin-Lang with *B. cinerea* isolate B05.10 [15].

Regions of PAV were investigated to identify potential missing genome regions in lupin-infecting isolates that are present in genomes of isolates infecting other hosts. Whole-genome alignments were performed using MUMmer v3.1 (nucmer, data-filter, show-snps, show-coords). The Sscl-Lang and Sscl-Lmut assemblies were compared to those of *S. sclerotiorum* 1980 [14], *S. borealis* [59] and *S. homoeocarpa* [58]. The Bcin-Lang assembly was compared to *B. cinerea* B05.10 (reference isolate) [15], *B. cinerea* T4 [6] and *B. cinerea* BcDW1 [60]. BEDTools CoverageBed [61] (intervals of 100 Kb) was used to calculate the percentage of the length of each sequence covered by one or more nucmer matches. Gene, repeat and G:C content for reference genomes were also calculated within these same intervals. The SNP and indel variants reported by MUMmer alignments were converted to GFF3 format and analysed for their effect on gene annotations via SnpEff v4.3, which calculated D_n_/D_s_ (count of non-synonymous over synonymous mutations) ratios. Match coverage, gene and repeat density, G:C content and D_n_/D_s_ ratios were visualized using Circos v0.69–6 [62]. Larger regions of sectional absence spanning multiple adjacent genes of the reference isolates versus one or more lupin-infecting isolates were identified where 3 or more adjacent genes covered < 30% by alignments of alternate isolate of the same species (Additional file 6).

PAV regions specific to lupin-infecting isolates were investigated to identify potential genes or genome regions specific to lupin-infection. Comparisons to the whole-chromosome reference assemblies were used to sort Bcin-Lang, Sscl-Lang and Sscl-Lmut contigs into “core” (conserved) and “non-core” (isolate-specific) sets. Contigs with MUMmer matches covering ≤30% of their length were considered isolate-specific, or “non-core”. Genes and functional annotations within these regions were manually inspected for potential roles in lupin pathogenicity and host-specificity (Additional file 2, Table 2).

## Additional files

Additional file 1: Statistically significant over−/under-representation of pfam functional annotations between pathogenic isolates or groups. (XLSX 31 kb)

Additional file 2: Information supporting effector prediction, orthology, functional annotations, mutations and PAV across all isolate gene sets. (XLSX 5734 kb)

Additional file 3: Comparison of the repeat contents of isolates of *Botrytis cinerea* and *Sclerotinia sclerotiorum*. (XLSX 13 kb)

Additional file 4: Information of genes associated with AT-rich regions identified in the lupin-infecting *Botrytis cinerea* isolate. (XLSX 11 kb)

Additional file 5: List of predicted effector genes in lupin-infecting isolates of *Botrytis cinerea* and *Sclerotinia sclerotiorum*. (XLSX 43 kb)

Additional file 6: Summary of PAV regions spanning 3 or more genes in reference isolates of *Botrytis cinerea* and *Sclerotinia sclerotiorum*, relative to alternate isolates of the same species. (XLSX 72 kb)

Additional file 7: Summary the relationship between repetitive sequences and PAV regions in reference isolates of *Botrytis cinerea* and *Sclerotinia sclerotiorum*. (XLSX 1065 kb)

Additional file 8: CAZyme profiles for lupin-infecting and reference isolates of *Botrytis cinerea* and *Sclerotinia sclerotiorum*. (XLSX 32 kb)

Additional file 9: Flow diagram of procedure used to exclude sequences from final assemblies due quality or contamination. (PDF 92 kb)

Additional file 10: List of fungal genome datasets used as evidence for alignments supporting in silico gene predictions. (XLSX 10 kb)

## References

[1] Gladstones J. Distribution, origin, taxonomy, history and importance. In: ‘Lupins as crop plants—biology, production and utilization’.(Eds JS Gladstones, C Atkins, J Hamblin) pp. 1–40. In. Cambridge: Cambridge University Press. p. 1998.

[2] Caballero B, Finglas P, Toldrá F (2015). Encyclopedia of food and health, 1st edn: academic.

[3] Clements JC, Wilson J, Sweetingham MW, Quealy J, Francis G (2012). Male sterility in three crop *Lupinus* species. Plant Breed.

[4] Western Australian lupin industry [https://www.agric.wa.gov.au/grains-research-development/western-australian-lupin-industry].

[5] White P, French B, McLarty A: Producing lupins. In*.* Edited by Department of Agriculture and Food, 2nd edn. Perth: South Perth, W.a. : Department of Agriculture and Food; 2008.

[6] Amselem J, Cuomo CA, Van Kan JA, Viaud M, Benito EP, Couloux A, Coutinho PM, De Vries RP, Dyer PS, Fillinger S (2011). Genomic analysis of the necrotrophic fungal pathogens *Sclerotinia sclerotiorum* and *Botrytis cinerea*. PLoS Genet.

[7] Boland G, Hall R (1994). Index of plant hosts of *Sclerotinia sclerotiorum*. Can J Plant Pathol.

[8] Maintenance of seed yield and quality in lupins in the presence of sclerotinia 2016 trial report [https://www.agric.wa.gov.au/lupins/maintenance-seed-yield-and-quality-lupins-presence-sclerotinia-2016-trial-report].

[9] Dean R, Van Kan JA, Pretorius ZA, Hammond-Kosack KE, Di Pietro A, Spanu PD, Rudd JJ, Dickman M, Kahmann R, Ellis J (2012). The top 10 fungal pathogens in molecular plant pathology. Mol Plant Pathol.

[10] Condon BJ, Leng Y, Wu D, Bushley KE, Ohm RA, Otillar R, Martin J, Schackwitz W, Grimwood J, MohdZainudin N (2013). Comparative genome structure, secondary metabolite, and effector coding capacity across *Cochliobolus* pathogens. PLoS Genet.

[11] van der Does HC, Rep M (2007). Virulence genes and the evolution of host specificity in plant-pathogenic fungi. Mol Plant-Microbe Interact.

[12] Jones DA, Bertazzoni S, Turo CJ, Syme RA, Hane JK (2018). Bioinformatic prediction of plant–pathogenicity effector proteins of fungi. Curr Opin Microbiol.

[13] Gibriel HA, Thomma BP, Seidl MF (2016). The age of effectors: genome-based discovery and applications. Phytopathology.

[14] Derbyshire M, Denton-Giles M, Hegedus D, Seifbarghy S, Rollins J, van Kan J, Seidl MF, Faino L, Mbengue M, Navaud O (2017). The complete genome sequence of the phytopathogenic fungus *Sclerotinia sclerotiorum* reveals insights into the genome architecture of broad host range pathogens. Genome biology and evolution.

[15] Van Kan JA, Stassen JH, Mosbach A, Van Der Lee TA, Faino L, Farmer AD, Papasotiriou DG, Zhou S, Seidl MF, Cottam E (2017). A gapless genome sequence of the fungus *Botrytis cinerea*. Mol Plant Pathol.

[16] Guyon K, Balagué C, Roby D, Raffaele S (2014). Secretome analysis reveals effector candidates associated with broad host range necrotrophy in the fungal plant pathogen *Sclerotinia sclerotiorum*. BMC Genomics.

[17] Heard S, Brown NA, Hammond-Kosack K (2015). An interspecies comparative analysis of the predicted secretomes of the necrotrophic plant pathogens *Sclerotinia sclerotiorum* and *Botrytis cinerea*. PLoS One.

[18] Buiate E, Xavier K, Moore N, Torres M, Farman M, Schardl C, Vaillancourt L (2017). A comparative genomic analysis of putative pathogenicity genes in the host-specific sibling species *Colletotrichum graminicola* and *Colletotrichum sublineola*. BMC Genomics.

[19] Chappell TM, Rausher MD (2016). Evolution of host range in *Coleosporium ipomoeae*, a plant pathogen with multiple hosts. Proc Natl Acad Sci.

[20] Parra G, Bradnam K, Korf I (2007). CEGMA: a pipeline to accurately annotate core genes in eukaryotic genomes. Bioinformatics.

[21] Testa AC, Oliver RP, Hane JK (2016). OcculterCut: a comprehensive survey of AT-rich regions in fungal genomes. Genome biology and evolution.

[22] Lu T, Yao B, Zhang C (2012). DFVF: database of fungal virulence factors. Database.

[23] Winnenburg R, Baldwin TK, Urban M, Rawlings C, Köhler J, Hammond-Kosack KE (2006). PHI-base: a new database for pathogen host interactions. Nucleic Acids Res.

[24] Pan Y, Wei J, Yao C, Reng H, Gao Z (2018). SsSm1, a Cerato-platanin family protein, is involved in the hyphal development and pathogenic process of Sclerotinia sclerotiorum. Plant Sci.

[25] Yang G, Tang L, Gong Y, Xie J, Fu Y, Jiang D, Li G, Collinge DB, Chen W, Cheng J (2018). A cerato-platanin protein SsCP1 targets plant PR1 and contributes to virulence of Sclerotinia sclerotiorum. New Phytol.

[26] Bertazzoni S, Williams A, Jones DA, Syme RA, Tan K-C, Hane JK. Accessories make the outfit: accessory chromosomes and other dispensable DNA regions in plant-pathogenic Fungi. Mol Plant-Microbe Interact. 2018; ja. 10.1094/MPMI-06-17-0135-FI 29664319

[27] Lo Presti L, López Díaz C, Turrà D, Di Pietro A, Hampel M, Heimel K, Kahmann R (2016). A conserved co-chaperone is required for virulence in fungal plant pathogens. New Phytol.

[28] Zerillo MM, Adhikari BN, Hamilton JP, Buell CR, Lévesque CA, Tisserat N (2013). Carbohydrate-active enzymes in *Pythium* and their role in plant cell wall and storage polysaccharide degradation. PLoS One.

[29] Seifbarghi S, Borhan MH, Wei Y, Coutu C, Robinson SJ, Hegedus DD (2017). Changes in the *Sclerotinia sclerotiorum* transcriptome during infection of *Brassica napus*. BMC Genomics.

[30] Hane JK, Oliver RP (2008). RIPCAL: a tool for alignment-based analysis of repeat-induced point mutations in fungal genomic sequences. BMC bioinformatics.

[31] Hane JK, Williams AH, Taranto AP, Solomon PS, Oliver RP. Repeat-induced point mutation: a fungal-specific, endogenous mutagenesis process. In: Genetic transformation Systems in Fungi, vol. 2: Springer; 2015. p. 55–68.

[32] Saupe SJ (2000). Molecular genetics of heterokaryon incompatibility in filamentous ascomycetes. Microbiol Mol Biol Rev.

[33] Atwell S, Corwin JA, Soltis NE, Subedy A, Denby KJ, Kliebenstein DJ (2015). Whole genome resequencing of *Botrytis cinerea* isolates identifies high levels of standing diversity. Front Microbiol.

[34] Chen W, Lee M-K, Jefcoate C, Kim S-C, Chen F, Yu J-H (2014). Fungal cytochrome p450 monooxygenases: their distribution, structure, functions, family expansion, and evolutionary origin. Genome biology and evolution.

[35] McHale L, Tan X, Koehl P, Michelmore RW (2006). Plant NBS-LRR proteins: adaptable guards. Genome Biol.

[36] Shelest E (2008). Transcription factors in fungi. FEMS Microbiol Lett.

[37] Lo Presti L, Lanver D, Schweizer G, Tanaka S, Liang L, Tollot M, Zuccaro A, Reissmann S, Kahmann R (2015). Fungal effectors and plant susceptibility. Annu Rev Plant Biol.

[38] Sonah H, Deshmukh RK, Bélanger RR (2016). Computational prediction of effector proteins in fungi: opportunities and challenges. Front Plant Sci.

[39] Porebski S, Bailey LG, Baum BR (1997). Modification of a CTAB DNA extraction protocol for plants containing high polysaccharide and polyphenol components. Plant Mol Biol Report.

[40] FastQC: a quality control tool for high throughput sequence data**.** [http://www.bioinformatics.babraham.ac.uk/projects/fastqc].

[41] Martin M (2011). Cutadapt removes adapter sequences from high-throughput sequencing reads. EMBnetjournal.

[42] Magoč T, Salzberg SL (2011). FLASH: fast length adjustment of short reads to improve genome assemblies. Bioinformatics.

[43] Bankevich A, Nurk S, Antipov D, Gurevich AA, Dvorkin M, Kulikov AS, Lesin VM, Nikolenko SI, Pham S, Prjibelski AD (2012). SPAdes: a new genome assembly algorithm and its applications to single-cell sequencing. J Comput Biol.

[44] Huson DH, Beier S, Flade I, Górska A, El-Hadidi M, Mitra S, Ruscheweyh H-J, Tappu R (2016). MEGAN community edition-interactive exploration and analysis of large-scale microbiome sequencing data. PLoS Comput Biol.

[45] RpeatMasker open-4.0 [http://www.repeatmasker.org].

[46] Huang X, Adams MD, Zhou H, Kerlavage AR (1997). A tool for analyzing and annotating genomic sequences. Genomics.

[47] Ter-Hovhannisyan V, Lomsadze A, Chernoff YO, Borodovsky M (2008). Gene prediction in novel fungal genomes using an ab initio algorithm with unsupervised training. Genome Res.

[48] Testa AC, Hane JK, Ellwood SR, Oliver RP (2015). CodingQuarry: highly accurate hidden Markov model gene prediction in fungal genomes using RNA-seq transcripts. BMC Genomics.

[49] Haas BJ, Salzberg SL, Zhu W, Pertea M, Allen JE, Orvis J, White O, Buell CR, Wortman JR (2008). Automated eukaryotic gene structure annotation using EVidenceModeler and the program to assemble spliced alignments. Genome Biol.

[50] Quevillon E, Silventoinen V, Pillai S, Harte N, Mulder N, Apweiler R, Lopez R (2005). InterProScan: protein domains identifier. Nucleic Acids Res.

[51] Yin Y, Mao X, Yang J, Chen X, Mao F, Xu Y (2012). dbCAN: a web resource for automated carbohydrate-active enzyme annotation. Nucleic Acids Res.

[52] Finn RD, Clements J, Eddy SR (2011). HMMER web server: interactive sequence similarity searching. Nucleic Acids Res.

[53] Bairoch A, Apweiler R (2000). The SWISS-PROT protein sequence database and its supplement TrEMBL in 2000. Nucleic Acids Res.

[54] Petersen TN, Brunak S, von Heijne G, Nielsen H (2011). SignalP 4.0: discriminating signal peptides from transmembrane regions. Nat Methods.

[55] Horton P, Park K-J, Obayashi T, Fujita N, Harada H, Adams-Collier C, Nakai K (2007). WoLF PSORT: protein localization predictor. Nucleic Acids Res.

[56] Sperschneider J, Catanzariti A-M, DeBoer K, Petre B, Gardiner DM, Singh KB, Dodds PN, Taylor JM (2017). LOCALIZER: subcellular localization prediction of both plant and effector proteins in the plant cell. Sci Rep.

[57] Sperschneider J, Gardiner DM, Dodds PN, Tini F, Covarelli L, Singh KB, Manners JM, Taylor JM (2016). EffectorP: predicting fungal effector proteins from secretomes using machine learning. New Phytol.

[58] Green R, Sang H, Chang T, Allan-Perkins E, Petit E, Jung G (2016). Draft genome sequences of the turfgrass pathogen *Sclerotinia homoeocarpa*. Genome announcements.

[59] Mardanov AV, Beletsky AV, Kadnikov VV, Ignatov AN, Ravin NV (2014). Draft genome sequence of *Sclerotinia borealis,* a psychrophilic plant pathogenic fungus. Genome announcements.

[60] Blanco-Ulate B, Allen G, Powell AL, Cantu D (2013). Draft genome sequence of *Botrytis cinerea* BcDW1, inoculum for noble rot of grape berries. Genome announcements.

[61] Quinlan AR, Hall IM (2010). BEDTools: a flexible suite of utilities for comparing genomic features. Bioinformatics.

[62] Krzywinski M, Schein J, Birol I, Connors J, Gascoyne R, Horsman D, Jones SJ, Marra MA (2009). Circos: an information aesthetic for comparative genomics. Genome Res.

