# Supplementary figures and images for: Prediction of pathogenicity genes involved in adaptation to a lupin host in the fungal pathogens Botrytis cinerea and Sclerotinia sclerotiorum via comparative genomics
Source: BMC Genomics. 2019 May 17;20:385. doi: 10.1186/s12864-019-5774-2 (PMC6525431; doi:10.1186/s12864-019-5774-2)

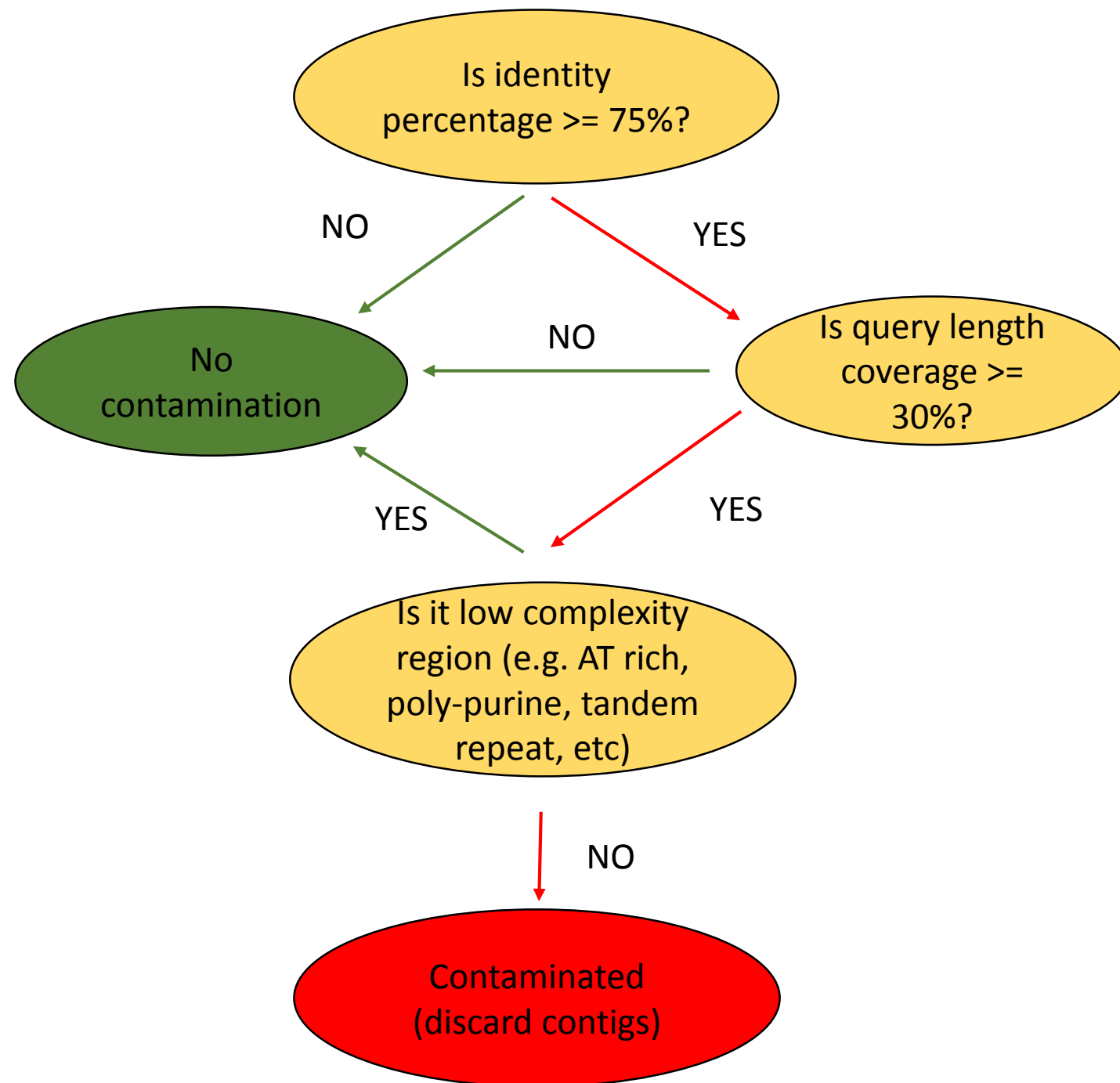

Supplement: Supplementary file 9 — Flow diagram of procedure used to exclude sequences from final assemblies due quality or contamination. (PDF 92 kb) [file 12864_2019_5774_MOESM9_ESM.pdf]
